# Tuberculosis Infection in the United States: Prevalence Estimates from the National Health and Nutrition Examination Survey, 2011-2012

**DOI:** 10.1371/journal.pone.0140881

**Published:** 2015-11-04

**Authors:** Roque Miramontes, Andrew N. Hill, Rachel S. Yelk Woodruff, Lauren A. Lambert, Thomas R. Navin, Kenneth G. Castro, Philip A. LoBue

**Affiliations:** Division of Tuberculosis Elimination, Centers for Disease Control and Prevention, Atlanta, Georgia, United States of America; Hospital San Agustín. Aviles. Asturias. Spain, SPAIN

## Abstract

**Background:**

Reexamining the prevalence of persons infected with tuberculosis (TB) is important to determine trends over time. In 2011–2012 a TB component was included in the National Health and Nutrition Examination Survey (NHANES) to estimate the reservoir of persons infected with TB.

**Methods:**

Civilian, noninstitutionalized U.S. population survey participants aged 6 years and older were interviewed regarding their TB history and eligibility for the tuberculin skin test (TST) and interferon gamma release assay (IGRA) blood test. Once eligibility was confirmed, both tests were conducted. Prevalence and numbers of TST positive (10 mm or greater), IGRA positive, and both TST and IGRA positive were calculated by adjusting for the complex survey design after applying corrections for item nonresponse and digit preference in TST induration measurements. To examine TST positivity over time, data from NHANES 1999–2000 were reanalyzed using the same statistical methods. The TST was performed using Tubersol, a commercially available purified protein derivative (PPD), rather than PPD-S, which was the antigen used in NHANES 1999–2000. Prior patient history of TB vaccination was not collected in this study nor were patients examined for the presence of a Bacillus of Calmette and Guerin (BCG) vaccine scar.

**Results:**

For NHANES 2011–2012, TST and IGRA results were available for 6,128 (78.4%) and 7,107 (90.9%) eligible participants, respectively. There was no significant difference between the percentage of the U.S. population that was TST positive in 2011–2012 (4.7% [95% CI 3.4–6.3]; 13,276,000 persons) compared with 1999–2000 (4.3%; 3.5–5.3). In 2011–2012 the percentage that was IGRA positive was 5.0% (4.2–5.8) and double TST and IGRA positivity was 2.1% (1.5–2.8). The point estimate of IGRA positivity prevalence in foreign-born persons (15.9%; 13.5–18.7) was lower than for TST (20.5%; 16.1–25.8) in 2011–2012. The point estimate of IGRA positivity prevalence in U.S.-born persons (2.8%; 2.0–3.8) was higher than for TST (1.5%; 0.9–2.6).

**Conclusions:**

No statistically significant decline in the overall estimated prevalence of TST positivity was detected from 1999–2000 to 2011–2012. The prevalence of TB infection, whether measured by TST or IGRA, remains lower among persons born in the United States compared with foreign-born persons.

## Introduction

In 2013, the World Health Organization estimated global tuberculosis (TB) rates at 1,260 cases annually per million population [1]. In the United States, TB rates have declined each year since 1993 and in 2013, an annual rate of 30 TB cases per million was reported [2]. The World Health Organization reports that the incidence of TB disease has been declining globally between 2000–2013 at a rate of 1.5% per year [1]. However, at the current rate of decline, achieving the internationally agreed upon target of global elimination of TB by 2050, defined as less than one case per million population per year, presents a formidable challenge [3–5]. A 2012 transmission model based on U.S. TB trend data indicated that elimination in the United States is not likely to be achieved by the end of the century [6]. While improvements in case detection and coordinated TB control efforts are improving globally, better point of care diagnostic capabilities, medication regimens, and service capacity are needed to accelerate progress toward global TB elimination [3].

Indiscriminate screening of persons at low risk for TB infection is not recommended as the positive predictive value of diagnostic tests for TB infection is low in this group. In addition, available diagnostic tests are unable to predict progression of infected individuals to disease, which means that large numbers of individuals need to be tested and treated to prevent an active and potentially infectious case [7, 8]. Additionally, screening of low-risk groups diverts resources away from higher priority TB control activities such as treatment of persons with TB disease and identifying and screening contacts of infectious cases [9–11].

In the United States preventing TB among U.S. and foreign-born persons infected with TB who have not yet developed disease is emphasized in addition to rapid diagnosis and treatment of persons with TB disease [12, 13]. However, TB infection is not symptomatic, transmissible, nor a reportable condition in many of the 50 U.S. states. Screening efforts are primarily focused on contacts of persons with infectious TB and other high-risk groups, including immigrants, refugees, incarcerated persons, and those experiencing homelessness [9, 12, 14–18].

Beginning in 1971, the Division of Tuberculosis Elimination of the U.S. Centers for Disease Control and Prevention (CDC) has periodically funded TB infection prevalence surveys included in the broader National Health and Nutrition Examination Survey (NHANES). NHANES is managed by CDC’s National Center for Health Statistics. The TB component was conducted to estimate the reservoir of persons infected with TB. In the absence of population-based surveillance, estimating the prevalence of persons infected with TB is important to examine trends over time in high-risk populations. The 1971 component of the survey was administered to only 35 of the 65 study sites. As a result, a smaller sample size was used to calculate the overall prevalence of TB infection (N = 1,580) limiting the precision of relevant demographic and socioeconomic estimates [19].

The 1999–2000 NHANES survey contained a TB component with a larger sample size (N = 7,613) while maintaining the methodology used in the 1971–1972 survey, which used tuberculin skin tests (TSTs) exclusively to test for TB infection [20]. The 1999–2000 survey also used TSTs, and results form the basis of the current comparison to the 2011–2012 NHANES survey results described in this manuscript. Additionally, an interferon gamma release assay (IGRA), not available for previous studies, was added to the 2011–12 survey to compare estimates of TB infection prevalence using two tests. Although comparisons of TST and IGRA test results for the diagnosis of TB infection have been conducted previously, the 2011–2012 NHANES survey is the first conducted on a civilian, non-institutionalized population based estimate of the United States [21–23].

## Methods

### Survey Methodology

NHANES is a series of sequentially run cross-sectional studies, implemented in 2-year cycles that assess the health of the civilian, noninstitutionalized U.S. population [24]. In order to obtain a nationally representative sample of the civilian, noninstitutionalized U.S. population, NHANES employs a complex, stratified, multistage probability cluster sampling design [25]. More than 5,000 persons participate in the survey in approximately 15 counties per year. Each survey participant is asked to respond to a series of health assessment questions in their home as the first step. Persons are subsequently asked to participate in a physical exam, which takes place in a mobile examination center specially equipped for this task. Survey questions and exam components may vary during each 2-year cycle as do requirements for testing a subset of persons based on factors such as age and race. In order to include a representative sample of the total noninstitutionalized U.S. population and increase the precision of estimates for certain subgroups, oversampling of Hispanic, non-Hispanic black, and Asians was done in 2011–2012. Additionally, white persons, at or below 130% of the federal poverty level, and persons over age 80 were oversampled [25]. NHANES participants provided informed consent and were provided monetary compensation for their involvement in the survey. This compensation was provided to all participants and was not unique to those taking part in the TB component.

The 2011–2012 TB component of NHANES included identical survey questions utilized during the 1999–2000 survey with the addition of one question that addressed treatment adherence for those persons previously treated for TB infection. For persons 6 years and older found to be eligible, each was given a tuberculin skin test (TST). Exclusion criteria included history of a severe reaction (i.e., anaphylactic shock or acute hypersensitivity reaction) to the purified protein derivative (PPD) solution used for the TST, or severe skin conditions such as burns or active eczema over both arms. If the participant was unable to return in the allotted time to read the TST, they were excluded. The TST was performed using a commercially available purified protein derivative (PPD) available from Sanofi Pasteur and incorporated under the trade name, Tubersol. Tubersol was used rather than PPD-S, the national standard PPD solution by which commercial tuberculin preparations are tested [26]. PPD-S was used in the 1999–2000 survey [27]. Although PPD-S is the U.S. standard tuberculin, each lot of commercially available Tubersol is tested for potency in comparison to PPD-S and is used by TB prevention programs throughout the United States and around the world [28]. Widespread availability and use made Tubersol an appropriate alternative to PPD-S, which is only available via an Investigational New Drug Protocol from the U.S. Food and Drug Administration.

For each participant, a trained phlebotomist or physician injected 0.1 mL (5 tuberculin units) of PPD into the volar surface of the right arm (or left arm, if the participant preferred). Staff documented which arm was used for the test. TST results were measured 46–76 hours later by trained TST readers who were not aware of the participant’s medical history or any history of contact with TB. Although the accepted standard is to read a TST result 48–72 hours after it has been placed [16, 29], a 46–76 hour time period was used to accommodate participant scheduling. This methodology was identical to NHANES 1999–2000 [27]. To increase the number of participants who had their TST results read, additional monetary compensation was provided, and readings were performed in a field office at the study site, or in the participant’s homes or workplaces, as necessary. TST readers measured TST results in a standardized way, by inspecting and palpating the surface of the forearm to determine if induration was present. If yes, the reader would mark the left and right borders of the indurated area transverse to the long axis of the forearm with an eyeliner pencil (for easy removal of the marks on the arm to make it blinded for the subsequent readers), and measure the induration size in millimeters using a standardized ruler [29]. Persons with an induration measured at 10 or greater millimeters were provided a form letter advising them to follow up with their physician or local health department for further evaluation.

In addition to receiving a TST, participants had blood drawn to screen for *Mycobacterium tuberculosis* infection using a commercially available immunologic test. The interferon gamma release assay (IGRA), QuantiFERON-TB Gold In-Tube (QFT-GIT; Cellestis/Qiagen, Carnegie, Victoria, Australia) was provided to all eligible patients 6 years of age or older. QFT-GIT is an in vitro diagnostic test that measures cell-mediated immune reactivity to *M*. *tuberculosis* antigens. The test is based on quantification of interferon-gamma released from sensitized lymphocytes when whole blood is incubated with control antigens and a cocktail of peptides representing three *M*. *tuberculosis* proteins, early secretory antigenic target-6 (ESAT-6), culture filtrate protein 10 (CFP-10), and TB 7.7. These proteins are in all *M*. *tuberculosis* but are absent from BCG vaccine strains and from most nontuberculous mycobacteria. As test antigens, these proteins offer the possibility of improved test specificity compared to PPD. QFT-GIT is an FDA-approved blood test for the detection of TB infection developed subsequent to the 1999–2000 survey [30, 31]. Persons who did not undergo phlebotomy as part of the NHANES exam were excluded. Study protocol required that blood be drawn for the QFT-GIT prior to placement of the TST.

All QFT-GIT results were conducted by a single Clinical Laboratory Improvement Act -certified laboratory during the duration of the 2011–2012 project period. Blood samples were drawn for the IGRA Assay from which plasma specimens were prepared and stored at the study site according to test recommendations and sent to the laboratory for testing the following day. Quantitative and qualitative values were reported for each QFT-GIT test result that was considered positive per the manufacturer and were based on the following criteria:

- Nil value must be ≤ 8.0 international units (IU) gamma interferon/ml AND
- TB antigen value minus Nil value must be ≤ 0.35 IU gamma interferon/ml AND
- TB antigen value minus Nil value must be ≤ 25% of the Nil value

Quality assurance was conducted throughout the survey periods 1999–2000 and 2011–2012 for TST placements and readings, and 2011–2012 for drawing blood for the QFT-GIT using standardized checklists [29]. At least two TST readers, blinded to each other’s measurements, read TST results of >25% of participants. Readers worked in separate rooms and recorded measurements in a computer database; measurements recorded by the first reader were not accessible to subsequent readers. An additional TST reading was done when CDC staff members were onsite to conduct quality assurance, which was done at least once per quarter during 2011–2012. For analysis purposes, NCHS provided the mean of up to three TST measurements for each participant who had more than one TST result recorded. The mean of TST results was also used in the NHANES 1999–2000.

As quality control for QFT-GIT, the laboratory repeated testing on a minimum of two percent of all specimens submitted. The repeat specimens were selected in a random manner using a number generator. All repeated result values were recorded and compared with initial results for concordance. Positive TST or QFT-GIT results indicated likely *M*. *tuberculosis* infection. Quarterly progress reports were generated and submitted to CDC’s National Center for Health Statistics (NCHS). The reports contained the number of specimens received and Statistical Analysis Quality Control results. Individual quality control results were not available to researchers for review and analysis.

### Statistical Analysis

Data analyses were performed with the open source statistical software R, version 3.1.1 [32] using the *survey* package [33]. Prevalence estimates for the U.S. population were based on Medical Examination Center sample 2-year weights [25], supplied in the NHANES dataset, which adjust for unequal probability of selection, unit nonresponse to the household interview and physical examination, and differences between the final sample and total population [34]. We further adjusted these weights to account for item nonresponse for TST, that is, a missing TST reading, using multivariate logistic regression to examine factors associated with latent TB infection (LTBI) and with not having a TST result in the dataset. New weights were produced by dividing the NHANES-supplied weight by the predicted probability of TST response within each category of covariates used in the logistic regression model for those Study Participants with a TST result. This was done in order to correct for possible bias in prevalence estimates based on those Study Participants with a TST reading. Details are S1 Text.

The *survey* package enables statistical inference which adjusts for the complex survey design. The NHANES survey design is described to R using the *svydesign* function and correct specification for variance estimation in subpopulations is implemented with the *subset* function. Weighted estimates for prevalence of TST positivity (TST ≥10 mm), IGRA positivity, and double positivity (TST ≥10 mm and IGRA positive) were derived using the *svyby* function which can calculate a variety of statistical measures. Proportions and their CIs are specified within this function with the *svyciprop* option and method = “logit”, vartype = “ci” calls. The “logit” method fits a logistic regression model and computes a Wald-type interval on the log-odds scale, which is back-transformed to the probability scale of 0 to 1.

Concordance of TST and QFT-GIT positivity was examined by using the *svytable* function to calculate 2-by-2 weighted crosstabulations of positive/negative proportions of these tests, adjusting for the survey design, to estimate joint outcomes of the two tests for the civilian, noninstitutionalized US population.

TST induration data for survey participants exhibited substantial digit preference, particularly a preference for 9 mm over 10 mm. TST induration data was, therefore, smoothed using a digit preference model, which was developed to incorporate previous approaches to dealing with digit preference [35, 36]. Our digit preference method identified proportions of each millimeter induration reading to be re-assigned to 1 millimeter either side of the original reading by smoothing. A single dataset, smoothed for TST positivity was constructed by randomly allocating the calculated proportion of millimeter re-assignments between 9 mm and 10 mm among survey participants. Multiple datasets were simulated in this way and the resulting point estimates of TST positivity were averaged to give an overall estimate. Variances were calculated as the sum of the average of the variances of datasets and the between-dataset variance of point estimates. Two-by-two concordance tables for TST and IGRA were similarly averaged over the multiple datasets. Details are in the S1 Text.

Numbers of persons with selected characteristics were estimated by multiplying prevalence estimates and lower and upper confidence intervals with corresponding U.S. Census Bureau population totals (S1 Text).

Estimation of LTBI prevalence for several strata may be unreliable due to small numbers of positives or large relative standard error (RSE). Following NCHS guidelines, we deemed to be unreliable those estimates which were based on fewer than 10 positives or for which the RSE was greater than 30%. Details of the RSE calculation are in the S1 Text.

To examine TST positive prevalence over time, data from NHANES 1999–2000 were reanalyzed by the same statistical methodology used for NHANES 2011–2012 survey data. Differences in TST positivity estimates between the two studies were assessed for statistical significance. Estimates from the two surveys were assumed to be statistically independent and a test for significant difference was conducted on the logit scale, assuming the logit estimates were normally distributed (S1 Text).

The NHANES protocol was reviewed by the National Center for Health Statistics’ Ethics Review Board and was deemed to be in compliance with Health and Human Services Policy for Protection of Human Research Subjects (45 CFR part 46, available from http://www.hhs.gov/ohrp/humansubjects/).

## Results

### Participation in NHANES Medical Examination, TST and IGRA

**Fig 1:**
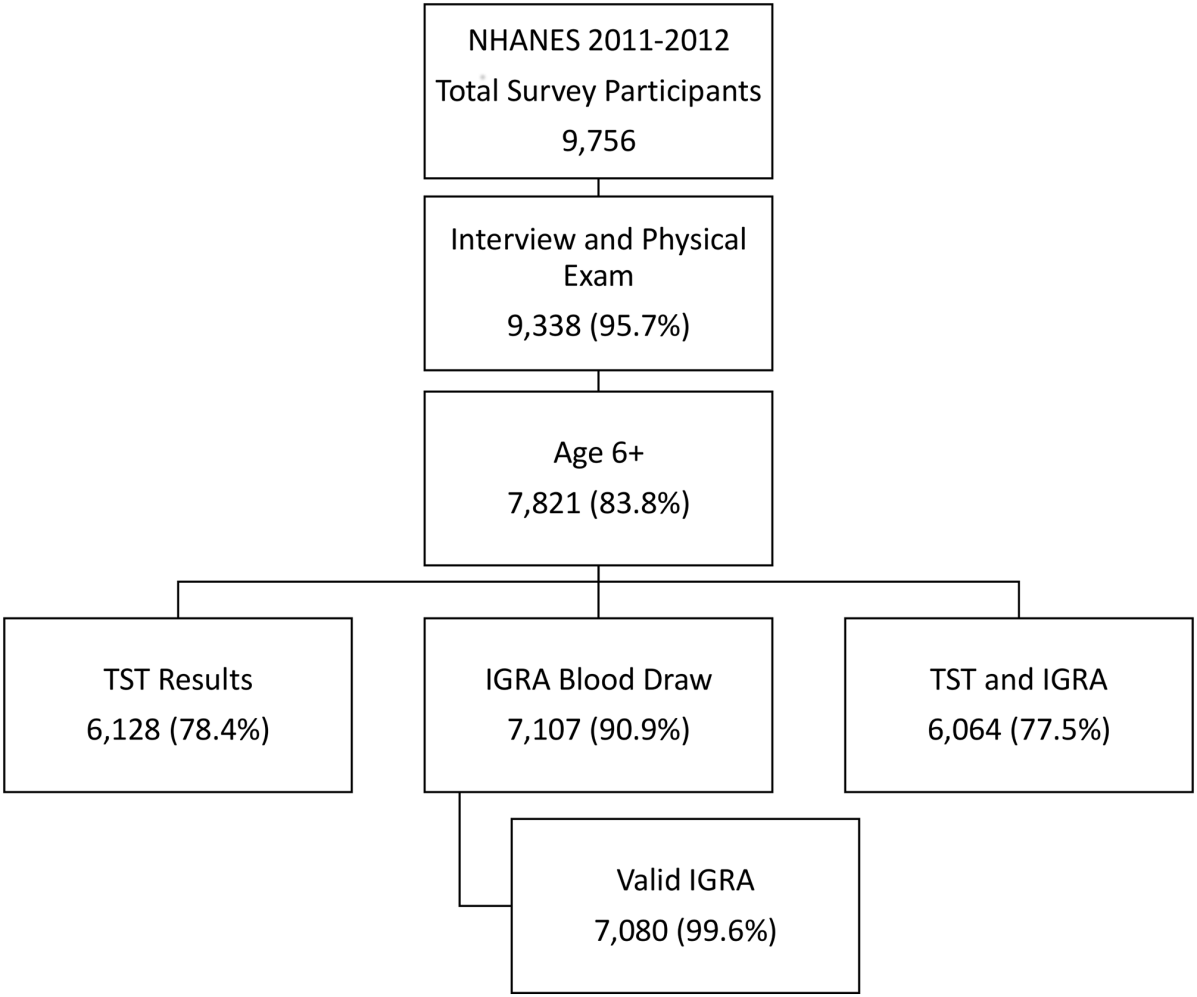
Flow chart illustrating participation in the 2011–2012 National Health and Nutrition Examination Survey, including total survey participants and number and percentage of survey participants who had tuberculin skin and interferon gamma release assay tests and results.

There were a total of 9,756 survey participants in the 2011–2012 NHANES (Fig 1). Of these, 9,338 (95.7%) had both a home interview and physical exam in the NHANES mobile examination center. Of the participants who had a physical exam after the interview, 7,821 (83.8%) were 6 years of age or older and eligible for the study; 6,128 (78.4%) had a TST placed and measured and 7,107 (90.9%) had blood drawn for IGRA. Valid (positive or negative) IGRA results were available for 7,080 (90.5%) eligible participants; 6,064 (77.5%) participants had both TST readings and valid IGRA results. Among participants who had at least one TST result, 59.8% had measurements recorded separately by two or more readers. In the 1999–2000 NHANES cycle 7,819 participants were 6 years of age or older; 6,679 (85.4%) had a TST placed and measured.

### Prevalence of Tuberculosis Infection Using TST

#### Overall

According to our weighted analysis, an estimated 4.7% of the civilian, noninstitutionalized U.S. population aged 6 years or older, or 13,276,000 persons, were infected with TB in 2011–2012 based on their TST response (Table 1). Among gender groups, 5.0% (95% CI 3.6–6.9) of males were infected and 4.4% (95% CI 3.2–5.9) of females were infected with TB. Among age groups, those aged 45–64 had the highest prevalence of infection, 6.3% (4.5–8.7). Among racial and ethnic groups, non-Hispanic Asians had the highest prevalence of TB, 22.2% (18.5–26.3). Among non-Hispanic Asians, Hispanics [12.3 (8.9–16.7)], and the foreign born [20.5% (16.1–25.8)] a prevalence of greater than 10% was observed. Cross-sectional 2011–2012 prevalence estimates along with 95% CIs for major subgroups are presented in Table 1. Comparable 1999–2000 prevalence estimates are included in Table 1 (data for non-Hispanic Asian and Hispanic groups were not available in NHANES 1999–2000). For the overall, U.S.-born and foreign-born populations, none was statistically different at the 0.05 level from the corresponding 2011–2012 estimates (respective *P*-values are 0.69, 0.48, 0.50).

**Table 1:** Tuberculin Skin Test Positive Prevalence in the Civilian, Noninstitutionalized U.S. Population, Ages 6+, 2011–2012 and 1999–2000.

	2011–2012		1999–2000	
Characteristics	TST Positive Prevalence, % (95% CI)	TST Positive, N (95% CI) x 1,000^a^	TST Positive Prevalence, % (95% CI)	TST Positive, N (95% CI) x 1,000^a^
Total	4.7 (3.4–6.3)	13,276 (9,604–17,795)	4.3 (3.5–5.3)	10,725 (8,730–13,220)
*Sex*				
Female	4.4 (3.2–5.9)	6,386 (4,644–8,563)	3.3 (2.3–4.6)	4,238 (2,954–5,907)
Male	5.0 (3.6–6.9)	6,866 (4,944–9,475)	5.4 (4.5–6.6)	6,534 (5,445–7,986)
*Age group*, *yr*				
6–14	1.0 (0.6–1.8)	370 (222–666)	1.2 (0.6–2.2)§	440 (220–807)
15–24	2.9 (1.8–4.7)	1,247 (774–2,021)	2.3 (1.2–4.5)§	877 (458–1,716)
25–44	5.7 (3.8–8.7)	4,590 (3,060–7,006)	4.8 (3.7–6.3)	3,957 (3,050–5,193)
45–64	6.3 (4.5–8.7)	5,157 (3,684–7,122)	6.4 (4.5–8.9)	3,818 (2,684–5,309)
≥65	4.3 (3.0–6.2)	1,724 (1,203–2,485)	5.4 (3.7–7.8)	1,757 (1,204–2,538)
*Race/ethnicity*				
Non-Hispanic white	1.0 (0.6–1.8)	1,821 (1,093–3,278)	2.0 (1.3–2.8)	3,575 (2,324–5,005)
Non-Hispanic black	6.9 (5.2–9.0)	2,323 (1,750–3,030)	7.7 (6.0–9.9)	2,370 (1,847–3,047)
Hispanic^b^	12.3 (8.9–16.7)	5,543 (4,010–7,525)	…	…
Non-Hispanic Asian^b^	22.2 (18.5–26.3)	3,057 (2,547–3,621)	…	…
*Poverty* ^c^				
At or above poverty	3.9 (2.7–5.6)	7,876 (5,453–11,309)	3.5 (2.7–4.5)	7,486 (5,775–9,625)
Below poverty	7.0 (5.6–8.7)	5,006 (4,005–6,222)	6.3 (4.3–9.2)	1,875 (1,280–2,738)
*Education level*				
<High school	6.0 (4.2–8.4)	4,910 (3,437–6,874)	5.6 (4.5–7.0)	2,542 (2,043–3,178)
High school graduate	4.8 (3.2–7.3)	3,184 (2,122–4,842)	3.4 (2.4–4.8)	2,248 (1,587–3,174)
Beyond high school	3.8 (2.8–5.0)	5,103 (3,760–6,715)	3.6 (2.4–5.3)	3,645 (2,430–5,366)
*Birthplace*				
United States	1.5 (0.9–2.6)	3,641 (2,185–6,312)	1.9 (1.5–2.5)	4,170 (3,292–5,487)
Foreign-born	20.5 (16.1–25.8)	8,139 (6,392–10,243)	18.1 (13.5–23.8)	5,421 (4,043–7,128)
*Diabetes* ^d^				
Normal	4.1 (3.0–5.6)	…	4.0 (3.1–5.1)	…
Prediabetes	6.7 (4.1–10.7)	…	9.1 (6.6–12.4)	…
Diabetes	8.7 (6.0–12.4)	…	6.9 (4.6–10.3)	…
*Body Mass Index* ^e^		…		…
Underweight	4.3 (1.6–11.0)§	…	1.4 (0.6–3.7)§	…
Normal	4.1 (2.9–5.8)	…	4.3 (3.1–6.0)	…
Overweight	5.0 (3.7–6.8)	…	5.2 (4.2–6.6)	…
Obese	5.1 (3.4–7.6)	…	4.3 (3.2–5.8)	…
*Cigarette Smoking Status*		…		…
Never Smoker	5.5 (3.9–7.7)	…	4.0 (2.7–6.0)	…
Former Smoker	5.3 (3.8–7.3)	…	6.2 (4.4–8.7)	…
Current Smoker	5.8 (3.6–9.4)	…	6.6 (4.9–8.9)	…

^a^ Calculated for characteristics for which a population denominator was available

^b^ Estimates for Hispanic and Asian subgroups cannot be calculated for 1999–2000 due to NHANES sampling methodology

^c^Defined using the ratio of family income to poverty (calculated by dividing family income by the U.S. Department of Health and Human Services poverty guidelines specific to the survey year); < 1 was considered below poverty

^d^ Normal, prediabetes and diabetes were defined using the National Institutes of Health’s glycohemoglobin (A1C) cutoff values http://diabetes.niddk.nih.gov/dm/pubs/diagnosis/

^e^ BMI categories for children and adolescents aged 2 to 19 years were based on the Centers for Disease Control and Prevention's sex-specific 2000 BMI-for-age growth charts for the United States http://www.cdc.gov/growthcharts/clinical_charts.htm. BMI for adults aged 20 years and older were based on Centers for Disease Control and Prevention’s standard weight status categories http://www.cdc.gov/healthyweight/assessing/bmi/adult_bmi/index.html

^§^ Estimates and 95% CIs may be unreliable because the RSE > 30%

Note: There were no study participants with both TST positive and HIV positive results

#### U.S.-born

**Fig 2:**
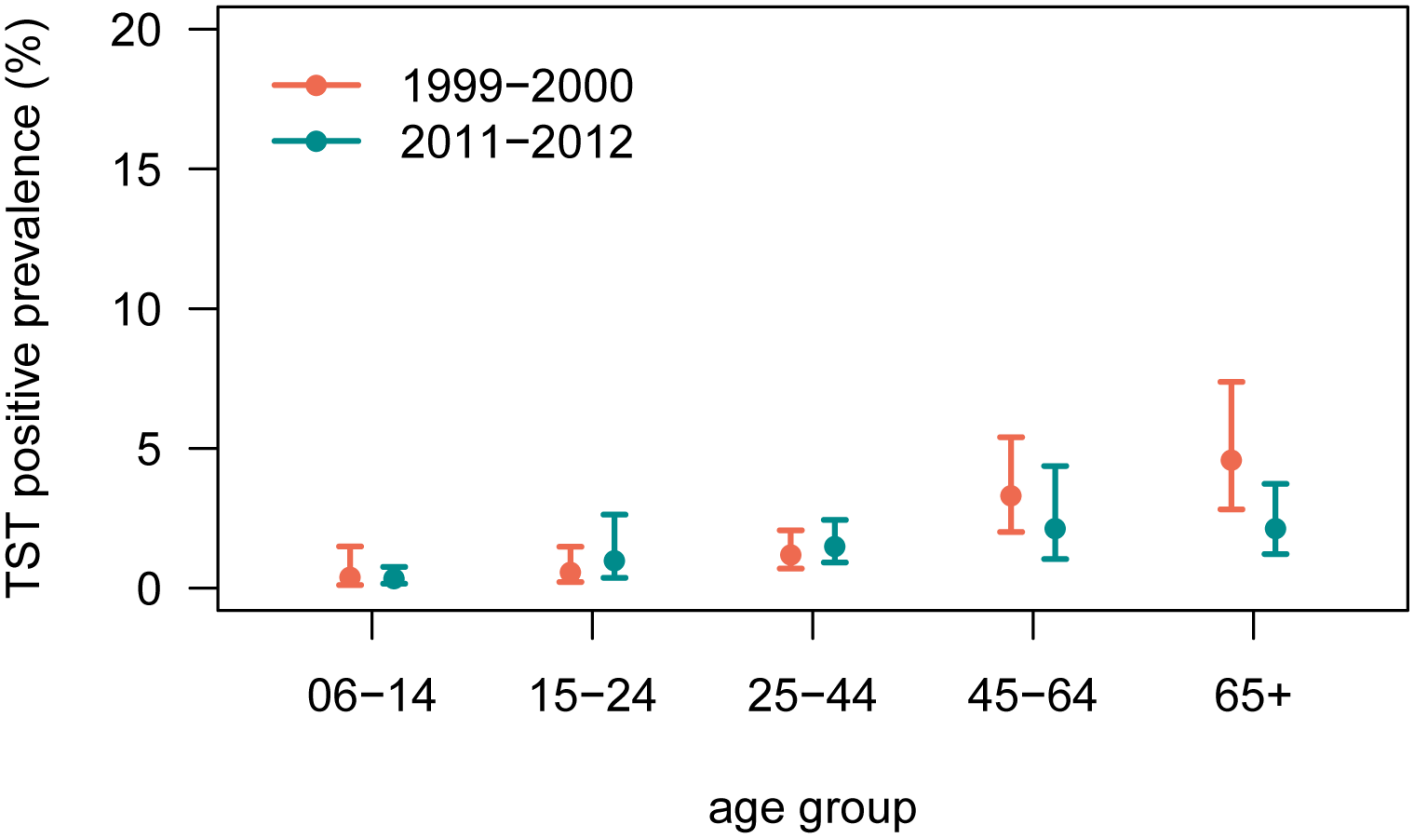
Tuberculin skin test positive prevalence estimates from the civilian, noninstitutionalized U.S.-born population aged 6 years or older, by age group. The orange points represent 1999–2000 prevalence estimates and the teal points represent 2011–2012 estimates. The vertical lines represent 95% confidence intervals.

TST positive prevalence among U.S.-born persons decreased slightly from 1.9% (1.5–2.5) in 1999–2000 to 1.5% (0.9–2.6) in 2011–2012 (Table 2). U.S.-born males had higher infection rates than females in both survey cycles. As age group increased, prevalence of TB infection increased to a peak of 2.1% (1.2–3.7) among persons aged 65 and older in 2011–2012; the increase in prevalence with age was more pronounced in 1999–2000 with a peak of 4.6% (2.8–7.4) in the 65 and older age group (Fig 2). The highest TB infection prevalence among racial and ethnic groups in 2011–2012 was observed among non-Hispanic Black persons [5.1% (3.6–7.3)]; all other racial and ethnic groups had TB infection prevalence rates below 3%.

**Table 2:** Tuberculin Skin Test Positive Prevalence in the Civilian, Noninstitutionalized U.S.-born Population, Ages 6+, United States, 2011–2012 and 1999–2000.

	2011–2012		1999–2000	
Characteristics	TST Positive Prevalence, % (95% CI)	TST Positive, N (95% CI) x 1,000	TST Positive Prevalence, % (95% CI)	TST Positive, N (95% CI) x 1,000
Total	1.5 (0.9–2.6)	3,641 (2,185–6,312)	1.9 (1.5–2.5)	4,170 (3,292–5,487)
*Sex*				
Female	1.3 (0.8–2.2)	1,622 (998–2,745)	1.5 (1.1–2.2)	1,697 (1,245–2,489)
Male	1.8 (1.0–3.1)	2,124 (1,180–3,658)	2.3 (1.5–3.5)	2,446 (1,595–3,722)
*Age group*, *yr*				
6–14	0.4 (0.2–0.8)* §	142 (71–284)	0.4 (0.1–1.5)§	139 (35–520)
15–24	1.0 (0.4–2.6)* §	388 (155–1,010)	0.6 (0.2–1.5)§	203 (68–507)
25–44	1.5 (0.9–2.4)	960 (576–1,536)	1.2 (0.7–2.1)	829 (484–1,451)
45–64	2.1 (1.0–4.4)§	1,458 (694–3,055)	3.3 (2.0–5.4)	1,730 (1,048–2,830)
≥65	2.1 (1.2–3.7)	734 (420–1,294)	4.6 (2.8–7.4)	1,357 (826–2,182)
*Race/ethnicity*				
Non-Hispanic white	0.7 (0.3–1.4)§	1,222 (524–2,445)	1.2 (0.7–2.0)	2,047 (1,194–3,411)
Non-Hispanic black	5.1 (3.6–7.3)	1,560 (1,101–2,233)	6.3 (4.7–8.3)	1,804 (1,346–2,377)
Hispanic^a^	2.9 (2.0–4.2)	771 (532–1,117)	…	…
Non-Hispanic Asian^a^	2.4 (0.9–5.9)* §	94 (35–232)	…	…

^a^ Estimates for Hispanic and Asian subgroups cannot be calculated for 1999–2000 due to NHANES sampling methodology

*Estimates and 95% CIs may be unreliable because the number of sample positives is < 10

^§^ Estimates and 95% CIs may be unreliable because the RSE > 30%

#### Foreign-born

**Fig 3:**
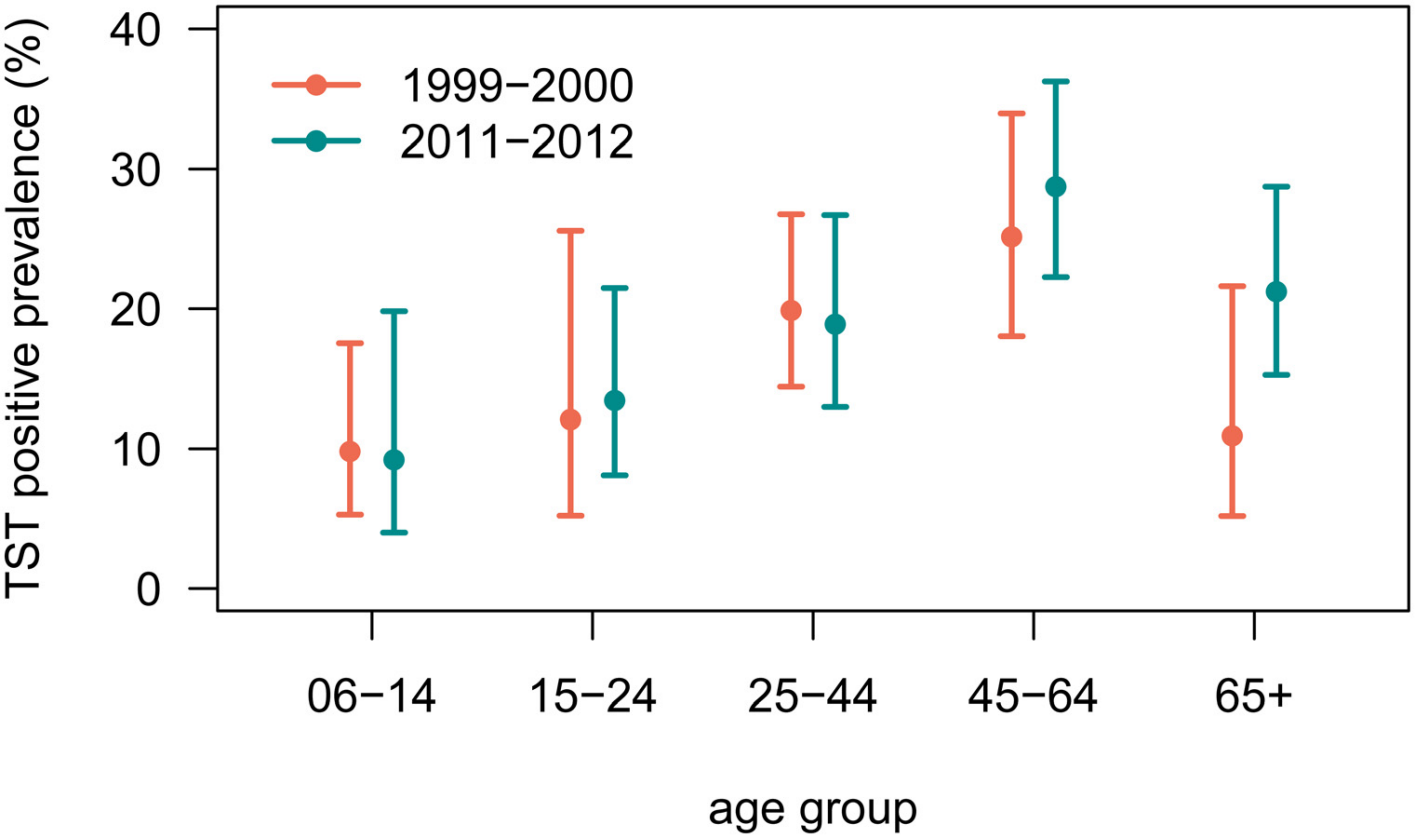
Tuberculin skin test positive prevalence estimates from the civilian, noninstitutionalized foreign-born population aged 6 years or older, by age group. The orange points represent 1999–2000 prevalence estimates and the teal points represent 2011–2012 estimates. The vertical lines represent 95% confidence intervals.

A disproportionate level of TB infection among foreign-born persons was observed in both NHANES 1999–2000 and 2011–2012 (Table 3). In 2011–2012, TB infection was distributed equally among males and females, 20.1 vs. 20.9%. The 45–64 age group had the highest prevalence of TB infection [28.8% (22.3–36.2)] although all age groups with the exception of 6–14 had a prevalence greater than 10% (Fig 3). Among racial and ethnic groups, a prevalence of greater than 20% was observed among non-Hispanic Asians [28.8% (24.7–33.4)], non-Hispanic Blacks [27.3% (19.8–36.3)], and Hispanics [20.3% (14.5–27.6)].

**Table 3:** Tuberculin Skin Test Positive Prevalence in the Civilian, Noninstitutionalized Foreign-born Population, Ages 6+, United States, 2011–2012 and 1999–2000.

	2011–2012		1999–2000	
Characteristics	TST Positive Prevalence, % (95% CI)	TST Positive, N (95% CI) x 1,000	TST Positive Prevalence, % (95% CI)	TST Positive, N (95% CI) x 1,000
Total	20.5 (16.1–25.8)	8,139 (6,392–10,243)	18.1 (13.5–23.8)	5,421 (4,043–7,128)
*Sex*				
Female	20.1 (15.4–25.8)	4,097 (3,139–5,259)	13.9 (8.8–21.4)	2,123 (1,344–3,269)
Male	20.9 (16.1–26.8)	4,037 (3,110–5,177)	21.9 (16.1–29.2)	3,214 (2,363–4,285)
*Age group*, *yr*				
6–14	9.2 (4.0–19.8)* §	135 (59–291)	9.8 (5.3–17.5)§	195 (106–348)
15–24	13.4 (8.1–21.5)	558 (337–896)	12.1 (5.2–25.6)§	522 (224–1,105)
25–44	18.9 (13.0–26.7)	3,122 (2,147–4,411)	19.9 (14.4–26.8)	2,658 (1,923–3,579)
45–64	28.8 (22.3–36.2)	3,579 (2,771–4,499)	25.2 (18.0–34.0)	1,823 (1,302–2,460)
≥65	21.2 (15.3–28.7)	1,085 (783–1,469)	10.9 (5.2–21.6)§	333 (159–659)
*Race/ethnicity*				
Non-Hispanic white	9.3 (4.2–19.3)§	696 (314–1,445)	16.7 (11.4–23.9)	1,369 (934–1,959)
Non-Hispanic black	27.3 (19.8–36.3)	838 (607–1,114)	20.1 (14.0–27.9)	430 (299–596)
Hispanic^a^	20.3 (14.5–27.6)	3,748 (2,677–5,096)	…	…
Non-Hispanic Asian^a^	28.8 (24.7–33.4)	2,833 (2,430–3,285)	…	…

^a^ Estimates for Hispanic and Asian subgroups cannot be calculated for 1999–2000 due to NHANES sampling methodology

* Estimates and 95% CIs may be unreliable because the number of sample positives is < 10

^§^ Estimates and 95% CIs may be unreliable because the RSE > 30%

### Alternative Definition of TB Infection: IGRA Positivity

In 2011–2012 an estimated 5.0% (4.2–5.8) of the civilian, noninstitutionalized U.S. population aged 6 years or older had IGRA positive results (Table 4) and 2.8% (2.0–3.8) of U.S.-born persons were IGRA positive compared to 15.9% (13.5–18.7) among foreign-born persons. The estimated numbers of TB-infected persons by definition of IGRA positivity is 14,123,000 (11,863,000–16,383,000) for the entire U.S. population. The estimated number of participants with positive IGRA results were similar among U.S.-born [6,797,000 (4,855,000–9,225,000)] and foreign-born [6,312,000 (5,360,000–7,424,000)] persons.

**Table 4:** Interferon Gamma Release Assay Positive Prevalence in the Civilian, Noninstitutionalized U.S. Population, Ages 6+, 2011–2012.

	IGRA Positive Prevalence, % (95% CI)		
Characteristics	Overall	U.S.-born	Foreign-born
Total	5.0 (4.2–5.8)	2.8 (2.0–3.8)	15.9 (13.5–18.7)
*Sex*			
Female	4.2 (3.3–5.3)	2.3 (1.4–3.6)	14.0 (11.3–17.3)
Male	5.8 (5.0–6.7)	3.3 (2.5–4.4)	17.9 (14.9–21.3)
*Age group*, *yr*			
6–14	0.9 (0.4–1.8)§	0.7 (0.3–1.7)§	2.6 (0.7–9.2)* §
15–24	3.0 (1.9–4.5)	2.2 (1.2–4.1)§	7.1 (3.3–14.4)§
25–44	4.4 (3.5–5.5)	1.9 (1.0–3.6)§	12.0 (9.3–15.5)
45–64	6.8 (5.1–8.9)	3.6 (2.3–5.6)	23.5 (18.5–29.4)
≥65	8.3 (6.5–10.5)	5.2 (4.0–6.9)	32.1 (24.4–40.9)
*Race/ethnicity*			
Non-Hispanic white	2.7 (1.9–3.7)	2.4 (1.6–3.5)	9.4 (4.6–18.5)§
Non-Hispanic black	5.3 (4.0–6.8)	4.4 (3.2–6.0)	15.2 (10.9–20.7)
Hispanic	10.2 (8.7–11.9)	3.7 (2.5–5.4)	15.6 (13.0–18.7)
Non-Hispanic Asian	17.5 (15.0–20.2)	2.9 (1.5–5.4)* §	22.3 (19.6–25.3)
*HIV Status* ^a^			
Negative	4.7 (3.8–5.7)	2.1 (1.3–3.3)	14.6 (12.1–17.4)
Positive	7.6 (3.3–16.7)* §	8.2 (3.3–19.1)* §	…

^a^ There were no foreign-born study participants with both IGRA positive and HIV positive results

* Estimates and 95% CIs may be unreliable because the number of sample positives is < 10

^§^ Estimates and 95% CIs may be unreliable because the RSE > 30%

IGRA positive prevalence followed similar patterns as TST positive prevalence with higher rates among males than females and increasing prevalence with increasing age group (with the exception of the 15–24 and 25–44 age groups among U.S.-born, which had IGRA prevalence rates of 2.2% (1.2–2.4) and 1.9% (1.0–3.6), respectively; Table 4). Furthermore, as with TST positive prevalence, non-Hispanic Asian persons had the highest IGRA positive prevalence among the overall population [17.5% (15.0–20.2)] and the foreign-born population [(22.3% (19.6–25.3)]. Among the U.S.-born, non-Hispanic Black persons had the highest prevalence of TB infection by IGRA [4.4% (3.2–6.0)].

### Alternative Definition of TB Infection: Double TST and IGRA Positivity

Applying an alternative definition of TB infection that required both TST and IGRA positivity yielded a prevalence of 2.1% (1.5–2.8) for the total population in 2011–2012 NHANES (Table 5). In the U.S.-born population alone, that prevalence was 0.6% (0.4–1.0) vs. 9.3% (7.4–11.7) among the foreign born. The use of this definition lowers the estimated number of TB-infected persons in the United States to 5,932,000 (4,293,000–7,796,000). Among the U.S. born that estimate is 1,457,000 (971,000–2,428,000) vs. 3,692,000 (2,938,000–4,645,000) in the foreign born.

**Table 5:** Tuberculin Skin Test and Interferon Gamma Release Assay Positive Prevalence in the Civilian, Noninstitutionalized U.S. Population, Ages 6+, 2011–2012.

	TST and IGRA Positive Prevalence, % (95% CI)		
Characteristics	Overall	U.S.-born	Foreign-born
Total	2.1 (1.5–2.8)	0.6 (0.4–1.0)	9.3 (7.4–11.7)
*Sex*			
Female	1.9 (1.4–2.6)	0.5 (0.3–1.0)§	9.1 (6.9–11.7)
Male	2.2 (1.6–3.1)	0.7 (0.4–1.2)	9.6 (7.2–12.6)
*Age group*, *yr*			
6–14	0.2 (0.1–0.8)* §	0.1 (0.0–0.5)* §	1.6 (0.2–10.2)* §
15–24	1.0 (0.4–2.3)* §	0.6 (0.1–2.8)* §	3.1 (1.3–6.9)* §
25–44	1.9 (1.2–3.0)	0.4 (0.2–0.7)*	6.6 (4.3–10.1)
45–64	3.2 (2.2–4.5)	0.7 (0.3–1.7)§	16.1 (12.4–20.7)
≥65	2.9 (1.9–4.3)	1.3 (0.7–2.3)§	15.2 (10.1–22.3)
*Race/ethnicity*			
Non-Hispanic white	0.3 (0.1–0.6)* §	0.2 (0.1–0.5)* §	2.8 (1.1–6.8)* §
Non-Hispanic black	3.1 (2.1–4.6)	2.5 (1.5–4.0)	10.5 (6.5–16.6)
Hispanic	5.8 (4.5–7.5)	1.1 (0.6–2.1)§	9.8 (7.6–12.4)
Non-Hispanic Asian	10.3 (8.0–13.3)	1.8 (0.6–4.9)* §	13.2 (10.6–16.3)

* Estimates and 95% CIs may be unreliable because the number of sample positives is < 10

^§^ Estimates and 95% CIs may be unreliable because the RSE > 30%

### Concordance between TST and IGRA

An estimated 94.5% of TST and IGRA test results were in agreement (Table 6). Test result discordance was higher among the foreign-born population (17.8%) compared to the U.S.-born population (3.1%). Among foreign-born persons with discordant test results, 11.2% had positive TST and negative IGRA results, and 6.6% had positive IGRA and negative TST results.

**Table 6:** Agreement between the Tuberculin Skin Test and Interferon Gamma Release Assay Test Results for Tuberculosis Infection in the Civilian, Noninstitutionalized U.S. Population, Ages 6+, 2011–2012.

	Overall Prevalence,%	U.S.-born Prevalence,%	Foreign-born Prevalence,%
TST Positive/IGRA Positive	2.1	0.6	9.3
TST Positive/IGRA Negative	2.6	0.9	11.2
TST Negative/IGRA Positive	2.9	2.2	6.6
TST Negative/IGRA Negative	92.4	96.3	72.9

### Survey Responses

Of 2011–2012 NHANES survey respondents, 64.2% (61.2–67.0) had previously been tested for TB infection. Among persons who had been tested previously, 5.9% (5.0–6.9) reported being told their test result was positive. Of those individuals, 43.7% (38.9–48.7) were prescribed medicine to prevent TB infection from progressing to TB disease and 91.7% (88.1–94.2) completed treatment. Only 0.4% (0.3–0.5) of survey respondents reported a previous diagnosis of TB disease, and 59.3% (36.3–78.9) of those individuals reported that they were prescribed medicine for treatment of TB.

## Discussion

Tuberculosis disease surveillance in the United States is robust and well documented [37]. However, TB infection results are not reported to the CDC. Since surveillance for TB infection in the United States is not currently done at the national level, prevalence surveys represent a relatively low-cost method to develop population estimates. This report represents the first national estimate of persons infected with TB disease since NHANES 1999–2000. In addition to presenting results of the analysis for NHANES 2011–2012, we re-analyzed 1999–2000 data to present a comparison among the overall population and major subgroups. Our analysis focused on TST results in order to directly compare with 1999–2000 TB infection prevalence, for which only TST results were available.

With the continued decline of TB disease in the United States and the country’s designation as a low TB-morbidity country, it was logical to posit that TB infection was declining from year-to-year. Furthermore, the birth cohort effect suggests that TB infection prevalence will decline among both U.S. and foreign-born populations as older birth cohorts, which typically have higher infection rates, are replaced with younger birth cohorts with lower rates of infection [38]. The current NHANES 2011–2012 survey results show no evidence of this presumed decline in infection prevalence over the last decade. In fact, there was a slight but statistically insignificant increase in the point estimate in the overall prevalence of TB infection from 1999–2000 to 2011–2012 (4.3% vs. 4.7%). Even if we were to assume the prevalence estimates from the two survey periods were highly positively correlated, resulting in a decreased standard error and a narrower confidence interval around the difference between the estimates, the confidence interval still contains zero and the difference remains statistically insignificant.

The point estimate increase in TB infection prevalence in the foreign-born population from NHANES 1999–2000 to 2011–2012 (18.1% vs. 20.5%) was also not statistically significant. This observed lack of decline stands in contrast to the trend in overall national TB disease rates, which declined during this period from 28.1 per 100,000 in 1999 to 15.9 per 100,000 in 2012 [37]. TB infection prevalence among foreign-born persons is influenced by TB infection rates in the country of origin [17, 39] and changing immigration patterns [17, 40]. It is possible, therefore, that a substantial drop in disease rates despite no decline in infection prevalence signifies effective TB control measures directed at foreign-born persons in the United States.

The NHANES survey population does not include those persons at highest risk of TB (incarcerated persons, persons experiencing homelessness, or elderly persons in long-term care facilities), and therefore TB infection prevalence data were not available among these groups. Exclusion of these persons from the survey likely resulted in an underestimation of the true prevalence overall and among the U.S.-born population [41–43]. Although HIV status was tested as part of NHANES, there were insufficient survey participants co-infected with TB infection and HIV to include in this analysis.

Defining positive TB infection using IGRA alone resulted in a slight increase compared with TST in the overall and U.S.-born prevalence estimates. However, the effect on foreign-born TB infection prevalence was more notable, decreasing that estimate to 15.9% (from 20.5% TST positive prevalence in 2011–2012). This is likely in part due to the absence of cross-reactivity to Bacillus Calmette-Guerin (BCG) vaccination resulting in higher false-positive TST results [31]; however we did not collect information about BCG for the 2011–2012 NHANES survey and were therefore unable to test this hypothesis using these national-level data. A rigorous statistical assessment of the difference in point estimates would have to take into account the amount of agreement between the tests, i.e., double positivity, as well as the complex survey design. The higher prevalence of IGRA positivity (2.8%) than TST positivity (1.5%) among the U.S.-born population was unexpected, and most [78.6%; (TST-/IGRA+) ÷ total IGRA+ = 2.2% ÷ (0.6%+2.2%); Table 6] of those with a positive IGRA had a negative TST. A similar study in military trainees in the United States reported that only 4 (0.3%) of 1546 low-risk trainees were IGRA positive and 3 (75%) of those were TST negative at a 10 mm cut off [44]. Among the U.S.-born population, an especially low-risk subgroup is non-Hispanic whites; in our analysis this group also had an unexpectedly high IGRA prevalence (2.4%), and most [91.7%; (IGRA+–TST+/IGRA+) ÷ IGRA+ = (2.4%– 0.2%) ÷ 2.4%; Tables 4 and 5] of these had a negative TST. Another study has demonstrated that the QFT-GIT assay can be associated with unexpected positive results when a low-risk population such as U.S. healthcare workers is screened and that these positive QFT-GIT results commonly revert to negative when the healthcare worker is retested with the same test [45].

Incorporating IGRA and TST into the definition for positive TB infection reduced the prevalence estimate by more than half (from 4.7% TST positivity to 2.1% combined TST and IGRA positivity). Similar reductions were seen in the prevalence point estimates among the U.S. and foreign-born populations. The double positive results represent a more conservative definition of TB infection than either test result alone, and if accurate would indicate a substantially lower TB infection prevalence rate than what we report based on TST positivity alone or IGRA positivity alone. However, CDC guidelines do not recommend routine use of both tests together. Additionally, steps should be taken to minimize unnecessary and misleading testing of persons at low risk [31].

While conventional, our choice of 10 mm cut-off is prone to bias from digit preference. For reasons unknown, the 2011–2012 data exhibited preference at 9 mm, whereas the more typical preference for 10 mm was present in the 1999–2000 data (S1 Text). We applied digit preference smoothing to both datasets so as to correct for underestimation of positive test results in the current dataset and overestimation in the older one. Crucially, this allows for comparison of prevalence estimates in each dataset to assess changes over the 12-year period between studies. This is because LTBI prevalence estimates for separate survey cycles will vary in accuracy depending on the criteria used to define TST positivity, whereas inference about the change in prevalence between successive survey cycles will be less affected by choice of criteria provided the same are used in both survey cycles. An examination of TST positivity with a >5 mm cut-off, which is considered positive in persons at high risk of developing TB, was further included in S1 Text to allow a comparison of this data to IGRA results.

IGRA blood test results are less subject to reader bias than TST [31]. Although questions about the interpretation of TST and IGRA results endure, agreement between the TST and IGRA tests was higher than previously published estimates [46, 47]. The IGRA component in the 2011–2012 NHANES may facilitate use of IGRA results as a measure of TB infection for trend analysis in the future.

This study contained a number of limitations. Tubersol was used in NHANES 2011–2012 rather than PPD-S, which was used in the 1999–2000 study to conduct the TST. This change in solutions had the potential to affect results making a comparison among the two studies less accurate. Although a random sample of at least two percent of QFT-GIT specimens were repeat tested for quality control, we were unable to review or analyze individual results as they were not available to researchers. This study did not collect information about BCG for the 2011–2012 NHANES survey. This limited our ability to examine cross-reactivity among vaccinated individuals and may have contributed to higher false-positive TST results.

Despite annual declines in TB disease case numbers and incidence rates, the NHANES survey detected no significant change in the rate of TB infection among the civilian, noninstitutionalized U.S. population since 1999–2000. The reservoir of TB infection in foreign-born persons, who comprised 65% of all reported cases of TB disease in 2013 [37], will continue to challenge efforts to decrease TB infection in the United States. The rates of TB disease among foreign-born persons are predicted to decline over the next 5 years [48]; all strategies for screening and treating foreign-born persons should be considered in order for TB infection measures to achieve a similar trend.

As positive predictive values of tests for low prevalence infections tend to be low, there will be ongoing challenges in estimating TB infection prevalence among the U.S.-born population. Furthermore, as the rate of TB disease declines among the U.S.-born population it will also become more important to target those known to be at higher risk of TB infection and disease, but not included in NHANES: incarcerated persons, persons experiencing homelessness, and elderly persons in long term care facilities. TB control efforts in the United States should focus on targeted testing of high-risk populations to make the most efficient use of resources.

## Supporting Information

S1 Text Online supplement to: Tuberculosis infection in the United States: Prevalence estimates from the National Health and Nutrition Examination Survey, 2011–2012. (DOCX)
