# Supplementary material for: Tuberculosis Infection in the United States: Prevalence Estimates from the National Health and Nutrition Examination Survey, 2011-2012
Source: PLoS One. 2015 Nov 4;10(11):e0140881. doi: 10.1371/journal.pone.0140881 (PMC4633161; doi:10.1371/journal.pone.0140881)
Supplement: S1 Text — (DOCX) [file pone.0140881.s001.docx]

ONLINE SUPPLEMENT TO

Tuberculosis infection in the United States: Prevalence estimates from the National Health and Nutrition Examination Survey, 2011-2012

Roque Miramontes, Andrew Hill, Rachel Yelk Woodruff, Lauren Lambert, Thomas Navin, Kenneth Castro, Philip LoBue

Methods

*Tuberculin Skin Testing*

Participants were eligible for the tuberculin skin test (TST) if they were at least 6 years of age, had completed the phlebotomy component of the NHANES medical examination, had never had a severe reaction to the tuberculin antigen, and were able to schedule a return visit between 46 and 76 hours after placement to have the skin test reaction measured. A trained phlebotomist or physician injected 0.1 ml of Tubersol® (a commercially available purified protein derivative available from Sanofi Pasteur) intradermally to the volar surface of the participant’s left forearm (or the right forearm if necessary) at least two inches from the elbow or wrist and away from veins, lesions, scars, heavy hair, tattoos, or other conditions that might make it difficult to measure the TST result [1]. Immediately following injection of the antigen, the placer used a millimeter ruler to measure the tense skin elevation, or wheal, created by the antigen. If the wheal was less than 6 millimeters in diameter, the placement was repeated in an area of the forearm at least 2 inches away from the first placement. The participant was instructed to avoid scratching or placing creams, lotions or medications on the injection site.

After proper placement of the TST, the placer made an appointment for the participant to have his/her skin test result measured. Every effort was made to schedule the return visit within 48-72 hours after administration of the skin test, and to only schedule in the 46-48 and 72-76 hour windows if no other option existed. No readings were permitted outside the 46-76 hour window. Measurements were conducted at the NHANES field office, or if necessary, at the participant’s home. Skin test readers made phone calls to participants to remind them of their scheduled appointments. If a participant missed his/her skin test reading appointment, the assigned reader attempted to contact the participant and reschedule the appointment before the 76-hour time limit was reached.

TST reactions were measured by trained NHANES TST readers who had no knowledge of the participant’s TB-related history. Readers worked in well-lit, private areas and followed a standard protocol for all TST measurements. The injection site was visually inspected and lightly palpated to differentiate between induration and soft swelling. If induration was present, the site was palpated to outline the margins (ridges) of induration, taking care not to confuse muscle ridges with induration margins. The induration margins were then marked using an eyebrow pencil at either side of the widest area of induration transverse to the long axis of the forearm. A millimeter ruler was placed such that the zero ruler line was inside the left dot edge, and the ruler was read at the line inside the right dot edge. If the measurement fell between two divisions on the millimeter scale, the lower mark was recorded. Dots were removed using baby oil and a gauze or cotton pad. A skin test report of findings was provided to each participant and included quantitative and qualitative results.

For quality control purposes, at least 25% of participants had their TST reactions measured by at least two separate readers who were blinded to each other’s results. Additionally, TST trainers from the Centers for Disease Control and Prevention (CDC) performed site visits to NHANES field offices once per quarter to ensure that standard TST reading protocol and techniques were being followed, and to determine if readers were meeting the goal of measuring results within 2 millimeters of CDC trainers and each other. If more than one reading was recorded when CDC trainers were not on site, the report of findings reflected the highest millimeter result; if a CDC trainer recorded a result that result was reported even if it was not the highest result recorded. For analysis purposes, the mean TST measurement was used for each participant who had more than one result recorded. Millimeter rulers used to measure TST reactions were marked by odd or even numbers (i.e. one set of rulers showed 10 mm, 20 mm, 30 mm, etc. and the other set of rulers showed 5 mm, 15 mm, 25 mm). The rulers were rotated by NHANES field staff so readers used rulers with even markings during one location and odd numbers during the next location.

*Blood Test*

The interferon gamma release assay (IGRA), QuantiFERON-TB Gold In-Tube (QFT-GIT; Cellestis/Qiagen, Carnegie, Victoria, Australia) was provided to all eligible patients 6 years of age or older. QFT-GIT is an in vitro diagnostic test that measures cell-mediated immune reactivity to *M. tuberculosis*. The test is based on quantification of interferon-gamma released from sensitized lymphocytes in whole blood incubated overnight with PPD from *M. tuberculosis* and control antigens.

Trained NHANES phlebotomists followed standardized NHANES venipuncture procedures for QFT-GIT blood collection [2]. Three QFT blood collection tubes (a Nil Control tube, TB Antigen tube and a Mitogen tube) were filled with 0.8 to 1.2 milliliters of blood; if a tube did not fill within this range another sample was obtained. After filling, the tubes were shaken up and down for 5 seconds (or approximately 10 times) to ensure proper mixing of the blood with the TB antigen. Tubes were incubated according to manufacturer instructions and shipped to the contract laboratory for testing.

After being received at the laboratory, the samples were checked and incubated at 37° C + 1° for 16 to 24 hours (unless this requirement was met at the collecting laboratory) [3]. After incubation, the tubes were centrifuged in order to separate the cells from the plasma. The tubes were then placed on an automated Dynex instrument, which runs the assay using Revelation DSX version 6.12, assay protocol DSX QFT 4pt ver4.12 output file. If the Dynex was not available, a backup manual procedure was followed [3]. Results were reported as positive, negative or indeterminate.

*Report of Findings and Referrals*

Participants with skin test results of 10 millimeters or more received a report that indicated they may have been infected with TB at some point in the past and provided with contact information for the local health department for further evaluation. NHANES TST readers were instructed to only provide information as written on the report of findings. If the participant had additional questions about test results, TB infection, or TB disease they were referred to a National Center for Health Statistics physician. All TST reports of findings indicated that the blood test for TB was still pending. Negative QFT results were included in the final NHANES results, which were mailed to participants 12-16 weeks following the examination; participants’ with positive QFT results were notified of their QFT results immediately via telephone.

*TB History*

The NHANES household interview, conducted prior to the medical examination, included questions related to participants’ history of being tested for TB, treated for TB, or exposed to someone with TB. The responses were self-reported; no verification or validation was done. Information from the interview was not available to NHANES staff involved in TST placement or measurement. The following TB-related questions were asked during the home interview:

- Have you ever been tested for TB?
- Which test did you receive – the needle just under the skin, a blood test or the tine test?
- Were you told that your skin test was positive for TB?
- Were you told that your blood test was positive for TB?
- Were you told that your tine test was positive for TB?
- After getting a positive TB test, were you prescribed any medicine to keep you from getting sick with TB?
- Did you complete this treatment?
- Were you ever told that you had active tuberculosis or TB?
- Were you ever prescribed any medicine to treat active tuberculosis or TB?
- Have you ever lived in the same household with someone while that person was sick with tuberculosis or TB?

*Numbers of Persons with Selected Characteristics*

Population data from the U.S. Census Bureau was used to calculate estimates of the number of persons with TB infection overall and by origin, sex, age group, race/ethnicity, poverty status and education. For the 1999-2000 NHANES survey cycle, population data were obtained from the December 1999 Current Population Survey [4]; population data for poverty status was obtained from the Current Population Survey’s 1999 March Supplement. The 2011 American Community Survey [5] was used to obtain population data for 2011-2012 NHANES survey cycle calculations. All population data were restricted to match the NHANES population of noninstitutionalized, civilian, U.S. population (and further restricted to ages 6 years and older to be consistent with the population eligible for TB testing). Population counts by sex, age and race/ethnicity matched those provided by NHANES (<http://www.cdc.gov/nchs/nhanes/response_rates_CPS.htm>). Numbers of persons with selected characteristics were calculated by multiplying the prevalence estimate and lower and upper confidence intervals with the appropriate population data for each characteristic of interest.

Statistical Methods

*Data Retrieval and Preparation, Software Used*

Data downloads and conversion from SAS export files were performed with R version 3.1.1 [6] using the *foreign* package. Data management, recodes, and derived variable creation were performed with base R. Statistical analyses, described in this supplement, were done with the *survey* package [7]. *Survey* package provides a comprehensive range of data analysis techniques for complex survey design, comparable to SUDAAN (Research Triangle Institute, Research Triangle Park, NC 27709-2194 USA). All statistical procedures took into account the complex survey design with pseudo-stratum variables, pseudo-primary sampling unit variables, and 2-year MEC weights by describing the survey design on the full dataset and domains (subpopulations) using the *svydesign* function. Correct specification for variance estimation in subpopulations is implemented with the *subset* function. This issue is known as domain estimation and it is vital to account for it in any complex survey design where subpopulations do not coincide with the strata, so as to produce correct standard errors.

*Adjustment of Weights for Item Non-Response (missing TST result)*

Prevalence estimates for the U.S. population were based on the NHANES 2011-2012 mobile examination center sample 2-year weights (WTMEC2YR) [8]. These estimates adjust for unequal probability of selection and non-response to the household interview and physical examination.

In NHANES 2011-2012, 9,338 survey participants (SPs) had both the home interview and physical examination conducted in the mobile examination center. TB examination and lab data were restricted to the 7,821 SPs 6 years of age or older, which corresponds to a target population of approximately 282.5 million. Of these SPs, TST measurements were available for 6,128 persons, which corresponds to approximately 228.0 million population if we use the NHANES-supplied MEC weights. Accordingly, we further adjusted the WTMEC2YR weights for item non-response, that is, a missing TST reading. Factors associated with latent TB infection (LTBI) and with not having a TST result in the dataset were examined using multivariate logistic regression with the *glm* function with family=binomial option. Predicted probabilities of response for TST measurements in categories (i.e., TST not missing) were calculated using the *fitted* function. New weights were produced by dividing the NHANES-supplied weight (WTMEC2YR) by the predicted probability of TST response within each category of covariates used in the logistic regression model for those SPs with a TST result. Otherwise, the new weight was set to zero. This was done in order to correct for possible bias in prevalence estimates based on those SPs with a TST reading. Potential predictor variables included demographic factors (e.g. age, gender, race/ethnicity, place of birth) and socioeconomic indicators (e.g. poverty status, educational attainment). After examining potential models, we selected a model that used age and place of birth as predictors. Hispanic race and ethnicity was significant as a predictor as well, but in the interests of retaining sufficiently large cell sizes for regression, we omitted it.

Following the example in Lumley’s text [9, p.191], we performed logistic regression on the unweighted survey data, as opposed to adjusting to the whole population using the complex survey design (*svyglm* function with family=quasibinomial option). This was done because item non-response in TST reading is a feature of the survey data itself and not of the overall U.S. population. Reweighting via a weighted logistic regression with *svyglm* would produce a population total based on the 6,128 SPs with TST reading of 282.5 million, coinciding with the number based on the full 7,821 SPs using the original NHANES weights. Our unweighted survey data based regression produced an estimate of 284.8 million, a relative difference of 0.07%. This amount of difference is sufficiently small that it did not significantly affect variance estimates based on our new weights. Moreover, National Center for Health Statistics guidelines [8] recommend that estimated population numbers for prevalence rates be based on American Community Survey denominators, and not those calculated from NHANES weights. As a result, 0.07% difference is minor. We demonstrate the close agreement obtained by using our new weights with NHANES weight-based demographic profiles for the population aged 6 and over (Supplement Table 1). The NHANES 1999-2000 sample weights were adjusted using this same method.

Although there was item non-response in IGRA results, it was not as pronounced as for TST: 714 out of 7,821 SPs were missing an IGRA result. Of these, 669 SPs were also missing TST reading. (There were 27 indeterminate IGRA readings as well.) We did not adjust for item non-response in IGRA results.

*Statistical Analyses*

In all analyses, the prevalence of TST and IGRA positivity, and associated 95% confidence intervals, were calculated using the *svyby* function with the *svyciprop* option and method=“logit”, vartype=“ci” calls. The “logit” call fits a logistic regression model and computes a Wald-type interval on the log-odds scale, which is back transformed to the interval (0, 1). The prevalence of TST, double positivity and 95% CIs, were derived from an imputation-like procedure which took into account digit preference at 9 mm induration. Imputation estimates were averaged and standard errors were based on the individual imputed standard errors, which were estimated with *svyciprop* and back-transformed from the logit scale to the probability scale. Details of imputation averaging are described below.

The relative standard error (RSE) was used as a measure of the reliability of statistical estimates. The RSE is the ratio of the standard error to the point estimate. Estimates were originally calculated on the logit scale, then back-transformed to the probability scale, according to the delta method (first order Taylor series) approximation. Therefore, the RSE was calculated as the ratio of the standard error on the probability scale, given by the delta method, to the point estimate on the probability scale. Specifically, if *p* denotes the probability point estimate and *s.e.* (logit) the standard error on the logit scale, then the delta method expression for the standard error on the probability scale is *p* × (1−*p*) × *s.e.*(logit) so that the RSE= (1−*p*) × *s.e.* (logit). Following NCHS recommendations, RSE > 0.3 was deemed to indicate an unreliable estimate.

*Tuberculin Skin Test Positive at 5 mm or Greater*

We examined TST positivity proportions resulting from a >5 mm cut-off (Table 2), which is considered positive in persons with recent close contact with TB or HIV infection.

*Digit Preference*

TST induration data for survey participants in the NHANES 2011-2012 survey cycle exhibited substantial digit preference, particularly a preference for 9 mm over 10 mm. Pronounced spikes were also apparent at 2 mm in the initial release of the induration data which comprised a single induration reading and also, though attenuated, in the subsequent updated release in early 2015 of the induration variable (TBDRUIND) which is an average of up to 3 induration readings per SP from different TST readers. (This variable was released for comparability with the NHANES 1999-2000 induration data and is given to 1 decimal place; for analysis, we rounded it down to the nearest whole integer mm reading.) These digit preferences were prominent across all clusters and strata and demographic variables. In this sense, the 2011-2012 induration data were qualitatively different from those in the 1999-2000 survey cycle which exhibited the more commonly observed slight preference for 10 mm over 9 mm. When defining latent TB infection prevalence as the proportion among all induration readings of those that are 10 mm or more, the preference for 2 mm is relatively unimportant, but that at 9 mm may lead to underestimation of LTBI.

Digit correction methods have been in currency for some time and in large part have been motivated by these problems with TST induration data [10]. We used a digit preference smoothing method to reduce the bias from the 9 mm preference. The method we employed is called the composite link model (CLM) [11] and is an extension of the methods in [10], based on the theoretical methods of [12]. All these methods adopt a penalized likelihood approach.

The composite link approach models a smooth, unobserved latent distribution of induration >0 mm and assumes that the observed induration readings follow Poisson distributions at each mm level. It models digit preference by redistributing the “true” latent distribution to the observed readings, via a mechanism that posits that the Poisson mean of each observed whole integer mm *x* are linear combinations of true readings at *x* – 1, *x*, and *x* + 1 mm. The coefficients of the linear combinations are the misreported proportions and allow determination of how much of each mm reading is transferred to its left or right neighbor or remains the same. While an alternate model could allow only misreporting for certain select mm values, when declaring the LTBI to be determined by a reading ≥ 10 mm, the redistributions at 8, 9, 10, 11, 12 mm constitute most of the effect and smoothing over all mm readings has minimal influence on the outcome for LTBI prevalence.

CLM necessarily leads to an underdetermined system of equations as there are 3 coefficients for readings from 2 mm to the second largest value observed and 2 coefficients for 1 mm and the maximum induration reading. To account for this, CLM adopts constrained weighted least squares regression with a penalty for smoothing which regulates the roughness of the estimated distribution. Additionally, CLM imposes an *L*_1_-penalty to constrain the misreporting proportions to lie between 0 and 1. The optimal fit is found by grid search over the 2 smoothing parameters corresponding to the penalties and minimizing the Akaike information criterion. CLM aims to select a small number of misreported proportions exhibiting the strongest effects, while shrinking the rest to zero. CLM fits were conducted using the R code generously and publicly provided by the lead author of [11] on his personal website [13].

We applied the CLM separately to the survey data for SPs by birthplace: for those born in the United States and for foreign-born. We did not attempt to incorporate smoothing with a regression procedure to predict which SPs had true 9 mm readings and which had true 10 mm readings as we wanted to allow for smoothing to either side of 9 and 10 mm as already described so as not so overestimate the misreporting fraction. Instead, we randomly assigned redistribution according to the misreporting transfers between 9 mm and 10 mm to create a digit corrected dataset. Among US-born SPs, the CLM estimated negligible transfer from 10 mm to 9 mm and predicted that 9 of 33 SPs with a 9 mm reading should be transferred to 10 mm; among foreign-born SPs, the CLM again estimated negligible transfer from 10 mm to 9 mm and predicted that 16 of 63 SPs with a 9 mm reading be transferred to 10 mm. Graphs of the smoothing procedure on the unweighted survey data are shown in Supplement Figure 1. Twenty such digit corrected datasets were created for both the US- and foreign-born datasets. These were adjusted for the complex survey design to extrapolate to the non-institutionalized US population aged 6 and above and to estimate TST positivity using the logit-method for proportions described previously. We adopted the techniques used in multiple imputation: from *m* estimated proportions $\hat{T}_{1}$, …, $\hat{T}_{m}$ with variances $v_{1},\ldots, v_{m}$ we computed the overall estimate as the mean $\bar{T}= \frac{1}{m}\sum_{j=1}^{m} \hat{T}_{j}$ and the overall variance, by taking into account variance between and within estimates, as $\hat{Var} \left[ \bar{T} \right]= \frac{1}{m}\sum_{j=1}^{m} v_{j}+\left( \frac{m+1}{m} \right) \frac{1}{m-1} \sum_{j=1}^{m} \left( \hat{T}_{j}-\bar{T} \right)^{2}$.

The same digit correction method was applied to the NHANES 1999-2000 survey cycle data.

*Comparison with other approaches to estimating LBI prevalence*

Determination of latent TB infection from an induration reading of 10 mm or greater is one of several commonly used criteria, including the definition of a positive TST result for persons who are contact to a TB case. Others include: 15 mm cut-off; 14 mm and above adjusted by a “sensitivity multiplier” of 1.22; or the frequency of 17 mm indurations plus twice the frequency of 18 mm and above (“mirror method”) [14]. From a different perspective, a Bayesian mixture method has been developed, which assumes that the probability density of induration readings comprises a mixture of three underlying distributions representing TB infection, cross-reactions arising from environmental mycobacteria and, if appropriate, BCG vaccination reactions [15]. Unlike the previously mentioned approaches, the mixture method is computationally intensive, but it is arguably less arbitrary than these others in that it doesn’t rely on more arbitrary cut-off points. Nevertheless, the epidemiological validity of its underlying assumptions has been disputed. The mixture method may be prone to interpretability problems, in that a lack of clear distinction between the environmental mycobacteria and BCG vaccination reaction components has been demonstrated for some induration datasets [16]. We decided not to apply the Bayesian mixture method for three reasons: unlike the 1999-2000 study, the 2011-2012 survey did not contain information on BCG status; to our knowledge, there has been no development of a theory that can incorporate the multistage complex sampling design of the NHANES survey and thus permit statistically rigorous variance calculations; and pronounced digit preference patterns in the 2011-2012 induration data would bias the estimation of the mixture distributions.

References

1. National Health and Nutrition Examination Survey. Tuberculosis skin test procedures manual. Atlanta, GA: Centers for Disease Control and Prevention, Jan 2011. Available: <http://www.cdc.gov/nchs/data/nhanes/nhanes_11_12/TBSkinTestManual.pdf>. Accessed 2014 Dec 16.
2. National Health and Nutrition Examination Survey. Laboratory procedures manual. Atlanta, GA: Centers for Disease Control and Prevention, Mar 2011. Available: <http://www.cdc.gov/nchs/data/nhanes/nhanes_11_12/2011-12_Laboratory_Procedures_Manual.pdf>. Accessed 2014 Dec 16.
3. University of Washington. Laboratory procedure manual. Seattle, WA: University of Washington, Sep 2013. Available from: <http://www.cdc.gov/nchs/data/nhanes/nhanes_11_12/Tuberculosis%20TB_QFT_met.pdf>. Accessed 2015 March 4.
4. Current Population Survey. Washington, DC: United States Census Bureau. Available: <https://www.census.gov/cps/>. Accessed 2014 July 7.
5. American Community Survey. Washington, DC: United States Census Bureau. Available: <https://www.census.gov/acs/www/about_the_survey/american_community_survey/>. Accessed 2014 Oct 24.
6. R Core Team (2014). R: A language and environment for statistical computing. R Foundation for Statistical Computing. Vienna, Austria. URL: <http://www.R-project.org/>.
7. T. Lumley Analysis of complex survey samples. J. Stat. Softw., 2004, 9(1): 1-19.
8. U.S. Centers for Disease Control and Prevention. National Health and Nutrition Examination Survey: Sample Design, 2011-2014. Available: <http://www.cdc.gov/nchs/data/series/sr_02/sr02_162.pdf> March, 2014.
9. T. Lumley. Complex Surveys: A Guide to Analysis Using R. John Wiley & Sons. Hoboken, NJ. 2010.
10. Eilers PHC, Borgdorff MW. Modeling and correction of digit preference in tuberculin surveys. Int. J. Tuberc. Lung Dis. 2004. 8(2): 232-9.
11. Camarda C, Eilers PHC, Gampe J. Modelling general patterns of digit preference. Stat. Model. 2008. 8(4): 385-401.
12. Eilers PHC. Ill-posed problems with counts, the composite link model and penalized likelihood. Stat. Model. 2007. 7(3): 239-54.
13. CG Camarda, Digit Preference Model. URL: https://sites.google.com/site/carlogiovannicamarda/r-stuff/digit-preference-model Accessed August 25, 2014.
14. Fine PE, Bruce J, Ponnighaus JM, Nkhosa P, Harawa A, Vynnycky E. Tuberculin sensitivity: conversions and reversions in a rural African population. Int J Tuberc Lung Dis. 1999 Nov;3(11):962-75.
15. Neuenschwander BE, Zwahlen M, Kim SJ, Lee EG, Rieder HL. Determination of the prevalence of infection with Mycobacterium tuberculosis among persons vaccinated against Bacillus Calmette-Guerin in South Korea. Am J Epidemiol. 2002 Apr 1;155(7):654-63.
16. Davies GR, Fine PE, Vynnycky E. Mixture analysis of tuberculin survey data from northern Malawi and critique of the method. Int J Tuberc Lung Dis. 2006 Sep;10(9):1023-9.

Supplement Table 1: Comparison of weight estimates for population percentages of demographic variables in the population ages 6+, 2011-2012 (mobile examination center).

|  | NHANES Weight | New Weight |
| --- | --- | --- |
| Sex |  |  |
| Female | 51.4 | 51.5 |
| Male | 48.6 | 48.5 |
| Age group, yr |  |  |
| 6-14 | 13.4 | 13.2 |
| 15-24 | 15.2 | 14.9 |
| 25-44 | 28.2 | 28.3 |
| 45-64 | 29.1 | 29.4 |
| >65 | 14.0 | 14.2 |
| Race/ethnicity |  |  |
| Non-Hispanic white | 63.9 | 63.6 |
| Non-Hispanic black | 12.2 | 12.0 |
| Hispanic | 16.0 | 17.2 |
| Non-Hispanic Asian | 5.1 | 4.6 |
| Other | 2.8 | 2.7 |
| Poverty |  |  |
| At or above poverty | 75.6 | 75.7 |
| Below poverty | 18.2 | 18.5 |
| Missing | 6.2 | 5.8 |
| Education |  |  |
| <High school | 32.0 | 32.0 |
| High school graduate | 16.9 | 17.2 |
| Beyond high school | 51.1 | 50.8 |
| Birthplace |  |  |
| United States | 83.8 | 83.6 |
| Foreign-born | 16.2 | 16.4 |

Supplement Table 2: Tuberculin Skin Test Positive (Defined as Induration of 5 mm or Greater) Prevalence in the Civilian, Noninstitutionalized U.S. Population, Ages 6+, 2011-2012

| Characteristics | TST Positive Prevalence,  % (95% CI) |
| --- | --- |
| All participants | 8.1  (6.1-10.8) |
| Sex |  |
| Female | 7.0  (5.1 -9.5) |
| Male | 9.4  (7.0-12.4) |
| Age group, yr |  |
| 6–14 | 3.5  (2.6 -4.7) |
| 15–24 | 5.6  (3.7 -8.4) |
| 25–44 | 10.4  (6.8-15.7) |
| 45–64 | 10.0  (7.6-13.0) |
| >65 | 6.8  (4.7 -9.6) |
| Race/ethnicity |  |
| Non-Hispanic white | 2.3  (1.6 -3.5) |
| Non-Hispanic black | 11.3  (8.8-14.4) |
| Hispanic | 20.7 (15.6-26.9) |
| Non-Hispanic Asian | 36.1 (30.5-42.2) |
| Other | 3.5  (1.3 -9.3) |
| Poverty^a^ |  |
| Poverty income index >1 | 7.4  (5.3-10.2) |
| Poverty income index < 1 | 10.5  (8.5-13.0) |
| Education level |  |
| <High school | 10.9  (7.9-15.0) |
| High school graduate | 8.4  (5.8-12.0) |
| Beyond high school | 6.3  (4.8 -8.2) |
| Birthplace |  |
| United States | 3.1  (2.0 -4.8) |
| Foreign-born | 33.7 (27.8-40.1) |
| Diabetes^b^ |  |
| Normal | 7.2  (5.2 -9.9) |
| Prediabetes | 11.3  (8.3-15.2) |
| Diabetes | 14.0 (10.5-18.4) |
| Body Mass Index^c^ |  |
| Underweight | 7.6  (3.8-14.7) |
| Normal | 7.8  (5.6-10.6) |
| Overweight | 8.6  (6.5-11.2) |
| Obese | 8.4  (5.9-11.8) |
| Cigarette Smoking Status |  |
| Never Smoker | 9.4  (6.9-12.5) |
| Former Smoker | 8.8  (6.2-12.4) |
| Current Smoker | 9.6  (6.5-14.1) |
| ^a^Defined using the ratio of family income to poverty (calculated by dividing family income by the U.S. Department of Health and Human Services poverty guidelines specific to the survey year); < 1 was considered below poverty  ^b^ Normal, prediabetes and diabetes were defined using the National Institutes of Health’s glycohemoglobin (A1C) cutoff values <http://diabetes.niddk.nih.gov/dm/pubs/diagnosis/>  ^c^BMI categories for children and adolescents aged 2 to 19 years were based on the Centers for Disease Control and Prevention's sex-specific 2000 BMI-for-age growth charts for the United States <http://www.cdc.gov/growthcharts/clinical_charts.htm>. BMI for adults aged 20 years and older were based on Centers for Disease Control and Prevention’s standard weight status categories <http://www.cdc.gov/healthyweight/assessing/bmi/adult_bmi/index.html> | |

Suppl. Fig. 1a: Digit smoothing, US-born, 2011-2012. Single reading (green); average (red).

Suppl. Fig. 1b: Digit smoothing, foreign-born, 2011-2012. Single reading (green); average (red). ****
